# Cognitive weaknesses or impairments on the NIH toolbox cognition battery in children and adolescents: base rates in a normative sample and proposed methods for classification

**DOI:** 10.3389/fpsyg.2025.1473095

**Published:** 2025-06-06

**Authors:** Nathan E. Cook, Grant L. Iverson, Justin E. Karr

**Affiliations:** ^1^Department of Physical Medicine and Rehabilitation, Harvard Medical School, Boston, MA, United States; ^2^Spaulding Rehabilitation Hospital, Charlestown, MA, United States; ^3^Mass General for Children Sports Concussion Program, Boston, MA, United States; ^4^Schoen Adams Research Institute at Spaulding Rehabilitation, Charlestown, MA, United States; ^5^Department of Psychology, University of Kentucky, Lexington, KY, United States

**Keywords:** cognition, neuropsychological tests, psychometrics, cognitive dysfunction, cognitive assessment, base rates, fluid intelligence

## Abstract

**Introduction:**

The National Institutes of Health Toolbox for Assessment of Neurological and Behavioral Function Cognition Battery (NIHTB-CB) is a brief neuropsychological battery for the assessment of crystalized (i.e., vocabulary and word reading) and fluid cognition (i.e., working memory, visual episodic memory, processing speed, and executive functions). This study examined the frequency of low NIHTB-CB scores and proposes flexible algorithms for identifying cognitive weaknesses and impairment among youth.

**Methods:**

Participants were 1,269 youth from the NIHTB-CB normative sample who did not have a neurodevelopmental, psychiatric, or medical problem that might be associated with cognitive difficulties (53% boys and 47% girls; M = 11.8 years-old, SD = 3.0, range 7–17). The sample included the following racial and ethnic composition: 58.1% White, 17.8% Black or African American, 16.8% Hispanic, 1.7% Asian, 3.1% multiracial and ethnic identities, and 2.6% not provided. The frequency of obtaining low scores falling at or below several cutoffs were calculated and stratified by gender, age, and crystalized intellectual ability.

**Results:**

Considering the five fluid tests, nearly two-thirds of children and adolescents obtained one or more scores ≤ 25th percentile, half obtained one or more scores ≤ 16th percentile, between a third and a fourth obtained one or more scores ≤ 9th percentile, and nearly a fifth obtained one or more scores ≤ 5th percentile. We propose flexible, psychometrically derived criteria for identifying a cognitive weakness or impairment.

**Discussion:**

Referencing the base rates of low scores will help researchers and clinicians enhance the interpretation of NIHTB-CB performance among children with cognitive weakness or impairments that are neurodevelopmental or acquired.

## 1 Introduction

Families often receive neuropsychology services for their child during challenging and trying times. For example, consider a child who sustains a traumatic brain injury during a tragic accident, such as being struck by a car while crossing the street. Thankfully, given advances in modern medicine, the child survives, though neuroimaging reveals extensive structural injury to the brain including contusions and hemorrhages. A brief neuropsychological evaluation early in the rehabilitation process could be pursued to document the extent of cognitive problems and in a serial fashion to monitor outcome. To this end, the current study joins considerable prior work aiming to refine the scientific approach to interpreting performances across tests in a neuropsychological battery and determine whether results indicate cognitive impairment or neurodevelopmental weakness. We contend that examining individual test scores without considering multivariate base rates (as described further below) may increase the risk of interpretive and diagnostic errors, which might negatively impact the quality of care clinical neuropsychology can offer patients and their families.

When administered a cognitive test battery, children and adolescents in the general population frequently obtain one or more low scores (Brooks and Iverson, 2010, 2012; Brooks et al., 2013a; Cook et al., 2019). When considered in the context of a test battery, obtaining one or more low scores, even at or below the 5th percentile (which might reasonably be interpreted as occurring relatively infrequently in isolation), is surprisingly common among healthy individuals who have no known clinical conditions. The number of low test scores healthy children obtain varies based on the length of the battery (i.e., the more tests administered and interpreted, the greater the base rate of low scores), the cutoff score used to define a *low* or *impaired* score (i.e., higher cut-offs, such as 1 *SD* below the mean result in greater base rate of low scores compared to lower cutoffs, such as 1.5 *SDs* below the mean), and the examinee’s overall level of intellectual functioning (e.g., higher levels of intelligence are associated with fewer obtained low scores) (Brooks, 2010; Brooks and Iverson, 2012; Brooks et al., 2009, 2010b; Cook et al., 2019).

The National Institutes of Health Toolbox for Assessment of Neurological and Behavioral Function Cognition Battery (NIHTB-CB) is a brief cognitive test battery (Weintraub et al., 2013). The battery is administered in an iPad-assisted format and includes seven tests assessing attention, working memory, visual episodic memory, language, processing speed, and executive functions. Tests are organized into composites scores of crystallized and fluid cognitive abilities as well as an overall total composite score. The developers evaluated and described the psychometric soundness of each test, including test–retest reliability (intra-class correlation coefficients ranging from 0.76 to 0.97) as well as convergent and discriminant validity (Bauer et al., 2013; Carlozzi et al., 2013; Gershon et al., 2013; Tulsky et al., 2013; Zelazo et al., 2013). Research on NIHTB-CB performances among children and adolescents has supported (i) a two-factor model of crystalized and fluid abilities (Akshoomoff et al., 2018), (ii) the convergent validity of fluid measures with traditional executive function tests (Kavanaugh et al., 2019), (iii) the usefulness of the NIHTB-CB as an outcome in clinical trials (Gladstone et al., 2019), and (iv) the feasibility of using the NIHTB-CB in pediatric rehabilitation settings (Watson et al., 2020).

The NIHTB-CB has been used extensively to examine the neuropsychological functioning of children and adolescents with diverse characteristics and clinical conditions. Examples of such conditions include preterm birth (Joseph et al., 2022); autism spectrum disorders (Jones et al., 2022; Solomon et al., 2021); fragile X syndrome, Down syndrome, and idiopathic intellectual disability (Dakopolos et al., 2024; Shields et al., 2023); congenital heart defects (Schmithorst et al., 2022; Siciliano et al., 2021; Wallace et al., 2024); diabetes (Shapiro et al., 2021); human immunodeficiency virus (Molinaro et al., 2021); epilepsy (Thompson et al., 2020); and mild traumatic brain injury (Chadwick et al., 2021). Moreover, researchers have also found that social/economic factors are associated with cognitive test performances, such that children from families of lower socioeconomic status obtain lower NIHTB-CB scores, on average (Hackman et al., 2021; Isaiah et al., 2023; Taylor et al., 2020). Thus, researchers and clinicians investigating and treating a broad array of clinical conditions as well as those investigating social and economic factors associated with pediatric health might consider using the NIHTB-CB. As the use of the NIHTB-CB continues to increase, investigators and clinicians alike would benefit from scientifically based methods for identifying cognitive weakness or impairment on the NIHTB-CB among children and adolescents that go beyond interpreting individual scores and that hold promise for reducing interpretive errors.

This study sought to examine the prevalence of low NIHTB-CB scores among youth from the battery’s normative sample to provide psychometrically based guidance for identifying potential cognitive impairment or neurodevelopmental weakness. The frequency of low scores among children and adolescents has been reported for other test batteries, such as those measuring intelligence (Brooks, 2010), executive functions (Brooks et al., 2013b; Cook et al., 2019), and memory (Brooks et al., 2009). Further, the base rates of low scores have been reported among children and adolescents on multidomain batteries, both using traditional paper-and-pencil neuropsychological tests (Brooks et al., 2010b) as well as computerized tests (Brooks et al., 2010a). For the NIHTB-CB, base rates of low scores among adults have been reported (Holdnack et al., 2017a; Holdnack et al., 2017b; Iverson and Karr, 2021; Karr et al., 2022a,b), but base rates have not been reported among children and adolescents. The purpose of this study was to (a) provide base rates for low scores among healthy youth on the NIHTB-CB, and (b) provide flexible algorithms for identifying cognitive weakness or impairment.

## 2 Methods

### 2.1 Participants

The NIHTB-CB normative sample (Gershon, 2016) includes 2,413 youth ranging in age from 7 to 17 years, of which 1,722 completed all seven tests. Participants were excluded if they had a self-or proxy-reported diagnosis or history of preexisting health conditions that may affect cognitive functioning including: (a) attention-deficit/hyperactivity disorder (*n* = 145); (b) specific learning disability (*n* = 51); (c) autism, Asperger’s syndrome, pervasive developmental disorder, or other autism spectrum disorder (*n* = 17); (d) developmental delay (*n* = 13); (e) mental health problems, including depression or anxiety (*n* = 53), bipolar disorder or schizophrenia (*n* = 3), a serious emotional disturbance (*n* = 5), or being hospitalized for emotional problems (*n* = 12); (f) alcohol or drug abuse (*n* = 2); (g) epilepsy/seizures (*n* = 12); (h) cerebral palsy (*n* = 2); (i) traumatic brain injury (*n* = 2); (j) head injury with loss of consciousness (*n* = 19) or head injury with amnesia (*n* = 3); (k) stroke or transient ischemic attack (*n* = 1); (l) brain surgery (*n* = 4); (m) diabetes (*n* = 11); (n) heart problems (e.g., heart attack, angina) (*n* = 12); (o) thyroid problems (e.g., Graves’ disease) (*n* = 9); or (p) hypertension (*n* = 6). Participants were also excluded if they had a self-or proxy-report of (q) being on an individualized education program (*n* = 76), (r) attending special education classes (*n* = 41), or (s) receiving private tutoring or schooling for learning problems (*n* = 27). A total of 453 children had *one or more* of the above exclusion criteria.

The final sample (*N* = 1,269) included 53.0% girls and 47.0% boys with a mean age of 11.8 years (*SD* = 3.0) and a mean maternal education of 12.6 years (*SD* = 2.5). The sample included the following racial and ethnic composition: 58.1% White, 17.8% Black or African American, 16.8% Hispanic, 1.7% Asian, 3.1% multiracial and ethnic identities, and 2.6% not provided. A subsample (*N* = 1,233) had sufficient data to calculate demographically adjusted scores, including 53.1% girls and 46.9% boys with a mean age of 11.8 years (*SD* = 3.0) and a mean maternal education of 12.7 years (*SD* = 2.4). The sample included the following racial and ethnic composition: 59.8% White, 18.2% Black or African American, 17.2% Hispanic, 1.6% Asian, 3.2% multiracial and ethnic identities, and 1.7% not provided. This study includes secondary analyses of deidentified data and was approved by the Institutional Review Board of Mass General Brigham (Protocol #: 2020P000504).

### 2.2 Measures

There are seven NIHTB-CB tests (Akshoomoff et al., 2013). The crystalized composite is based on the Picture Vocabulary and Oral Reading Recognition tests (Gershon et al., 2013). The fluid composite is based on the other five tests: List Sorting Working Memory (Tulsky et al., 2013), Picture Sequence Memory (Bauer et al., 2013), Pattern Comparison Processing Speed (Carlozzi et al., 2013), Flanker Inhibitory Control and Attention (Zelazo et al., 2013), and the Dimensional Change Card Sort (Zelazo et al., 2013). Detailed descriptions of all tests are provided elsewhere (Holdnack et al., 2017b) and can also be accessed at: https://www.nihtoolbox.org/domain/cognition/. Age-adjusted scores are normed as Standard Scores (SS; *M* = 100, *SD* = 15) and demographically adjusted scores are normed as T-scores (*M* = 50, *SD* = 10), adjusting for age, gender, maternal education, and race/ethnicity based on published formulas (Casaletto et al., 2015).

### 2.3 Statistical analyses

The crystallized composite score, which consists of a receptive vocabulary test and a single word reading test, was used as a proxy for overall intellectual ability consistent with prior research on the NIHTB-CB (Holdnack et al., 2017b). This composite was either age-or demographic-adjusted, depending on this analysis, meaning the score reflected the expected language ability for a typically developing child of similar age or demographic characteristics. Tests of vocabulary and verbal abilities have been examined as potential predictors of children’s overall intellectual functioning or general cognitive ability (Schoenberg et al., 2007). Further, single word reading tests have long been used as estimates of overall intellectual abilities in adults, though less attention has been paid to children (Davis et al., 2018). In a sample of healthy children, the NIHTB-CB crystallized composite score correlated highly with composite scores of language abilities (r = 0.90) and fluid cognitive abilities (e.g., tests of attention, processing speed, working memory, and executive functions) (r = 0.71), showing a strong correspondence between this score and performances on several cognitive domains (Akshoomoff et al., 2013). Given prior evidence that the base rate of low cognitive test scores is strongly associated with level of intellectual ability (Brooks, 2010; Brooks and Iverson, 2012; Brooks et al., 2009; Brooks et al., 2010b; Cook et al., 2019), we examined the base rates of low test scores on the five fluid subtests stratified by crystalized composite standard scores. Specifically, the base rates of low scores among the five fluid scores was calculated across the following cutoffs: (a) *≤25th percentile* was defined as SS ≤ 90 and T ≤ 43; (b) *≤16th percentile* was defined as SS ≤ 85 and T ≤ 40; (c) *≤9th percentile* was defined as SS ≤ 80 and T ≤ 36 (N.B., there is no whole number T score that corresponds to the 9th percentile, and a T score of 36 was selected as this cutoff, because it corresponds to the lowest whole number T score typically interpreted as unusually low in clinical practice); (d) *≤5th percentile* was defined as SS ≤ 76 and T ≤ 34; and (e) *≤2nd percentile* was defined as SS ≤ 70 and T ≤ 30. These percentile cutoffs for defining low scores are consistent with previous research (Brooks et al., 2013a; Cook et al., 2019; Karr et al., 2017, 2018). The frequencies of age-adjusted low scores were stratified by gender, age bands (i.e., 7–8, 9–10, 11–12, 13–14, 15–17), and crystalized composite standard scores (i.e., ≤89, 90–99, 100–109, ≥110). The frequencies of demographically adjusted low scores were stratified by crystalized composite T scores (i.e., ≤43, 44–49, 50–56, ≥57).

Descriptive interpretive labels were provided for the frequencies of low scores to aide the translation to clinical practice. The number of low scores obtained by the top performing 75% of the standardization sample reflected *Broadly Normal* performance; the number of low scores obtained by fewer than 25% of the standardization sample reflected *Below Average* performance; the number of low scores obtained by fewer than 10% of the standardization sample reflected *Uncommon* performance; and the number of low scores obtained by less than 3% of the standardization sample reflected *Very Uncommon* performance. For age-adjusted norms, these qualitative ranges are provided for the full sample and stratifications by gender, age, maternal education, and crystalized ability. For demographically adjusted norms, these descriptive labels are provided for the full sample and stratifications by crystalized ability.

Flexible algorithms were developed to identify patterns of performances on the NIHTB-CB involving two or more low scores at various cutoffs that may be interpreted as a cognitive weakness or cognitive impairment. They were influenced by previously published studies of the NIHTB-CB adult normative sample (Holdnack et al., 2017b; Iverson and Karr, 2021; Karr et al., 2022b). Algorithms based on patterns of performances that occur in 16% or fewer (i.e., 1 *SD* below the mean) and 7% or fewer (i.e., 1.5 *SDs* below the mean) were created. A pattern that occurred in roughly 16% of the normative sample was selected based on DSM-5 psychometric diagnostic criteria for mild neurocognitive disorder, which requires a standardized cutoff of approximately 1 *SD* below the mean on neuropsychological testing for defining cognitive impairment (American Psychiatric Association, 2013). Because the frequencies of low scores varies by crystalized ability, separate algorithms for defining cognitive impairment were developed for different stratifications of crystalized composite scores.

## 3 Results

When considering the five fluid tests, a substantial percentage of healthy children and adolescents from the NIHTB-CB normative sample obtained low scores based on various cutoffs (see Table 1). Roughly two-thirds of children and adolescents obtained one or more scores ≤ 25th percentile (age-adjusted norms: 62.4%; demographically adjusted norms: 65.2%), about half obtained one or more scores ≤ 16th percentile (age-adjusted norms: 45.1%; demographically adjusted norms: 47.1%), between a third and a fourth obtained one or more scores ≤ 9th percentile (age-adjusted norms: 29.1%; demographically adjusted norms: 24.9%), and nearly a fifth obtained one or more scores ≤ 5th percentile (age-adjusted norms: 15.9%; demographically adjusted norms: 16.3%).

**Table 1:** Base rates of low scores on five NIHTB-CB fluid subtests stratified by gender, age, and crystalized ability level.

	Age-adjusted norms												Demographically-adjusted norms				
	TotalSample			Age					Crystalized composite				TotalSample	Crystalized composite			
		Boys	Girls	7–8	9–10	11–12	13–14	15–17	≤89	90–99	100–109	≥110		≤43	44–49	50–56	≥57
Sample size	1,269	597	672	220	260	258	235	296	301	305	339	324	1,233	307	300	301	325
≤25th percentile																	
5 low scores	0.9	1.5	0.4	0.5	1.2	1.9	0.9	0.3	2.0	1.3	0.6	–	0.8	1.3	1.3	0.7	–
4 or more	4.4	5.7	3.3	2.3	5.8	6.6	3.4	3.7	8.3	5.6	2.9	1.2	4.3	6.5	5.7	3.7	1.5
3 or more	13.3	13.7	12.9	7.3	20.0	14.7	11.1	12.5	20.6	15.7	10.6	7.1	14.9	22.5	18.0	10.3	9.2
2 or more	33.0	36.7	29.8	32.7	40.0	32.6	27.2	32.1	48.2	35.4	27.1	22.8	33.5	45.6	38.7	27.2	23.1
1 or more	62.4	65.2	60.0	67.7	67.3	64.7	54.9	58.1	76.1	64.3	58.7	51.9	65.2	75.6	69.3	59.8	56.6
No low scores	37.6	34.8	40.0	32.3	32.7	35.3	45.1	41.9	23.9	35.7	41.3	48.1	34.8	24.4	30.7	40.2	43.4
≤16th percentile																	
5 low scores	0.2	0.3	0.1	–	0.4	0.8	–	–	0.7	0.3	–	–	0.2	0.3	0.3	–	–
4 or more	1.3	1.5	1.0	0.5	0.4	2.3	0.9	2.0	3.0	1.3	0.6	0.3	1.5	2.3	2.7	1.3	–
3 or more	5.0	5.2	4.8	2.7	3.1	7.8	4.3	6.4	10.0	4.3	3.5	2.5	5.4	8.5	7.3	4.3	1.5
2 or more	16.8	17.8	15.9	13.2	15.8	16.7	15.3	21.6	26.9	17.7	14.2	9.3	17.1	25.4	19.0	12.6	11.7
1 or more	45.1	48.1	42.4	45.5	41.9	47.7	42.1	47.6	62.8	45.6	39.8	33.6	47.1	56.4	53.0	39.9	39.7
No low scores	54.9	51.9	57.6	54.5	58.1	52.3	57.9	52.4	37.2	54.4	60.2	66.4	52.9	43.6	47.0	60.1	60.3
≤9th percentile																	
5 low scores	0.1	0.2	–	–	–	0.4	–	–	–	0.3	–	–	0.1	–	0.3	–	–
4 or more	0.3	0.3	0.3	0.5	–	0.4	0.4	0.3	1.0	0.3	–	–	0.2	–	0.3	0.3	–
3 or more	1.7	1.5	1.8	0.5	1.2	2.7	2.1	1.7	3.7	1.0	1.2	0.9	1.2	2.0	2.0	0.7	0.3
2 or more	6.8	7.7	6.0	4.5	6.2	6.2	10.2	6.8	13.3	6.9	5.0	2.5	4.4	9.1	4.3	2.7	1.5
1 or more	29.1	31.7	26.8	29.1	27.3	29.1	32.3	28.0	44.9	29.5	23.3	20.1	24.9	36.2	27.7	19.9	16.3
No low scores	70.9	68.3	73.2	70.9	72.7	70.9	67.7	72.0	55.1	70.5	76.7	79.9	75.1	63.8	72.3	80.1	83.7
≤5th percentile																	
5 high scores	0.1	0.2	–	–	–	0.4	–	–	–	0.3	–	–	–	–	–	–	–
4 or more	0.2	0.2	0.1	–	–	0.4	–	0.3	0.3	0.3	–	–	0.1	–	0.3	–	–
3 or more	0.3	0.3	0.3	–	–	0.8	0.4	0.3	1.0	0.3	–	–	0.4	0.3	1.0	–	0.3
2 or more	2.4	2.7	2.2	1.4	1.2	3.5	3.4	2.7	5.0	2.3	2.1	0.6	2.3	4.2	3.0	1.0	0.9
1 or more	15.9	17.3	14.7	17.7	16.9	18.6	13.6	13.2	29.2	14.8	11.8	9.0	16.3	24.8	18.0	12.6	10.2
No low scores	84.1	82.7	85.3	82.3	83.1	81.4	86.4	86.8	70.8	85.2	88.2	91.0	83.7	75.2	82.0	87.4	89.8
≤2nd percentile																	
3 or more	0.1	–	0.1	–	–	–	–	0.3	0.3	–	–	–	–	–	–	–	–
2 or more	0.6	0.7	0.4	–	–	0.4	0.9	1.4	1.3	0.3	0.6	–	0.6	0.7	1.0	0.7	0.3
1 or more	7.1	9.2	5.2	7.7	6.5	8.1	6.4	6.8	14.3	3.9	8.0	2.5	6.4	10.1	7.0	4.7	4.0
No low scores	92.9	90.8	94.8	92.3	93.5	91.9	93.6	93.2	85.7	96.1	92.0	97.5	93.6	89.9	93.0	95.3	96.0

NIHTB-CB, National Institutes of Health Toolbox for the Assessment of Neurological and Behavioral Function Cognition Battery. All values represent cumulative percentages except for the rows labeled “No low scores,” which provide the percentage of the normative sample with no scores falling below the low score cut-offs. Age norms are provided as age-corrected standard scores (M = 100, SD = 15) and fully demographic corrected norms are provided as T scores (M = 50, SD = 10) adjusted for age, sex, maternal education level, and race/ethnicity. The NIHTB-CB includes five tests of fluid abilities: Flanker Inhibitory Control and Attention Test, Picture Sequence Memory Test, List Sorting Working Memory Test, Dimensional Change Card Sort Test, and Pattern Comparison Processing Speed Test. Two tests measure crystalized abilities, from which the crystalized composite is calculated: Picture Vocabulary Test and Oral Reading Recognition Test.

### 3.1 Low scores stratified by crystalized ability

**Figure 1:**
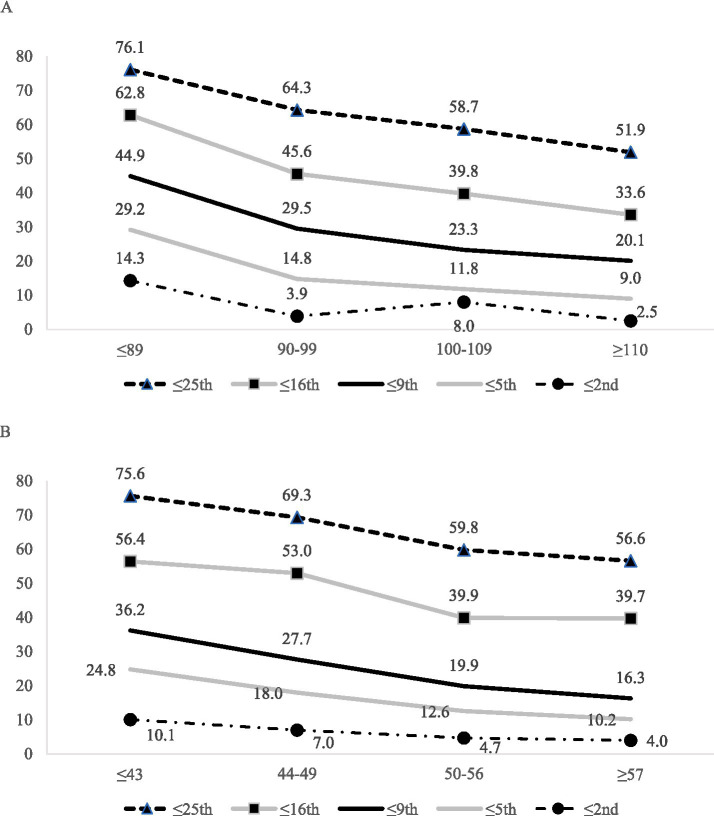
Base rates of one or more low fluid subtest scores across various cutoff scores stratified by NIHTB-CB crystalized composite (5 fluid scores were used in base rate calculations). **(A)** Base rates of one or more low age-adjusted fluid subtest scores across various cutoff scores stratified by NIHTB-CB age-adjusted crystalized composite (5 fluid scores were used in base rate calculations). **(B)** Base rates of one or more low demographically adjusted fluid subtest scores across various cutoff scores stratified by NIHTB-CB demographically adjusted crystalized composite (5 demographically adjusted fluid scores were used in base rate calculations).

Youth with low crystallized composite scores are more likely to obtain low fluid test scores (see Table 1 and Figure 1). For example, considering age-adjusted norms, one or more scores ≤ 25th percentile was obtained by 76.1% of youth with a below average crystalized composite (i.e., SS ≤ 89), compared to 51.9% of youth with an above average crystalized composite (i.e., SS ≥ 110). Similarly, considering demographically adjusted norms, one or more scores ≤ 25th percentile were obtained by 75.6% of youth with a below average crystalized composite (i.e., T ≤ 43) compared to 56.6% of youth with an above average crystalized composite (i.e., T ≥ 57). The clinical implications of this association are more notable at more stringent cutoffs. For example, only about 1 in 10 youth with above average crystalized composites obtained any scores ≤ 5th percentile (age-adjusted norms: 9.0%; demographically adjusted norms: 10.2%), whereas nearly 1 in 3 youth with below average crystalized composites obtained at least one score this low (age norms: 29.2%; demographically adjusted norms: 24.8%).

### 3.2 Classification ranges for low scores

Classification ranges for the expected number of low scores are provided (see Table 2), which can serve as an interpretive guide for the overall pattern of performances. For instance, using age-adjusted norms, obtaining two or more scores ≤ 16th percentile is considered *Broadly Normal* for youth with below average crystalized composite scores (i.e., SS ≤ 89), *Below Average* for youth with average crystalized composite scores (i.e., SS = 90–109), and *Uncommon* for youth with above average crystalized composite scores (i.e., SS ≥ 110). Similarly, it would be considered *Broadly Normal* for a healthy adolescent with a demographically adjusted crystalized composite in the average range (i.e., T = 44–56) to obtain two low scores ≤ 25th percentile, but this would be considered *Below Average* for an adolescent with a crystalized composite score in the above average range (i.e., T ≥ 57).

**Table 2:** Classification ranges for number of low NIHTB-CB fluid subtest scores for children and adolescents.

	Age-adjusted norms												Demographic-adjusted norms				
	Total Sample			Age					Crystalized composite				Total Sample	Crystalized composite			
		Boys	Girls	7–8	9–10	11–12	13–14	15–17	≤89	90–99	100–109	≥110		≤43	44–49	50–56	≥57
Sample size	1,269	597	672	220	260	258	235	296	301	305	339	324	1,233	307	300	301	325
≤25th percentile																	
Broadly normal	0–2	0–2	0–2	0–2	0–2	0–2	0–2	0–2	0–2	0–2	0–2	0–1	0–2	0–2	0–2	0–2	0–1
Below average	3	3	3	–	3	3	3	3	3	3	3	2	3	3	3	3	2
Uncommon	4	4	4	3	4	4	4	4	4	4	–	3	4	4	4	4	3
Very uncommon	5	5	5	4–5	5	5	5	5	5	5	4–5	4–5	5	5	5	5	4–5
≤16th percentile																	
Broadly normal	0–1	0–1	0–1	0–1	0–1	0–1	0–1	0–1	0–2	0–1	0–1	0–1	0–1	0–2	0–1	0–1	0–1
Below average	2	2	2	2	2	2	2	2	3	2	2	–	2	–	2	2	2
Uncommon	3	3	3	–	3	3	3	3	4	3	3	2	3	3	3	3	–
Very uncommon	4–5	4–5	4–5	3–5	4–5	4–5	4–5	4–5	5	4–5	4–5	3–5	4–5	4–5	4–5	4–5	3–5
≤9th percentile																	
Broadly normal	0–1	0–1	0–1	0–1	0–1	0–1	0–1	0–1	0–1	0–1	0	0	0	0–1	0–1	0	0
Below average	–	–	–	–	–	–	2	–	2	–	1	1	1	–	–	1	1
Uncommon	2	2	2–3	2	2	2	–	2	3	2	2	–	2	2	2	–	–
Very uncommon	3–5	3–5	4–5	3–5	3–5	3–5	3–5	3–5	4–5	3–5	3–5	2–5	3–5	3–5	3–5	2–5	2–5
≤5th percentile																	
Broadly normal	0	0	0	0	0	0	0	0	0–1	0	0	0	0	0	0	0	0
Below average	1	1	1	1	1	1	1	1	–	1	1	–	1	1	1	1	1
Uncommon	–	–	–	–	–	2	2	–	2	–	–	1	–	2	2	–	–
Very uncommon	2–5	2–5	2–5	2–5	2–5	3–5	3–5	2–5	3–5	2–5	2–5	2–5	2–5	3–5	3–5	2–5	2–5
≤2nd percentile																	
Broadly normal	0	0	0	0	0	0	0	0	0	0	0	0	0	0	0	0	0
Below average	–	–	–	–	–	–	–	–	1	–	–	–	–	1	–	–	–
Uncommon	1	1	1	1	1	1	1	1	–	1	1	–	1	–	1	1	1
Very uncommon	2–5	2–5	2–5	2–5	2–5	2–5	2–5	2–5	2–5	2–5	2–5	1–5	2–5	2–5	2–5	2–5	2–5

NIHTB-CB, National Institutes of Health Toolbox for the Assessment of Neurological and Behavioral Function Cognition Battery. Age norms are provided as age-corrected standard scores (SS; M = 100, SD = 15) and fully demographic corrected norms are provided as T scores (M = 50, SD = 10) adjusted for age, sex, maternal education level, and race/ethnicity. The NIHTB-CB includes five tests of fluid abilities: Flanker Inhibitory Control and Attention Test, Picture Sequence Memory Test, List Sorting Working Memory Test, Dimensional Change Card Sort Test, and Pattern Comparison Processing Speed Test. Two tests measure crystalized abilities, from which the crystalized composite is calculated: Picture Vocabulary Test and Oral Reading Recognition Test. The classification ranges in the first column are as follows: Broadly normal: this number of low scores were obtained by the top 75% of the normative sample; Below average: this number of low scores were obtained by less than 25% of the normative sample; Uncommon: this number of low scores were obtained by less than 10% of the normative sample, and Very uncommon: this number of low scores were obtained by less than 3% of the normative sample.

### 3.3 Algorithms for defining cognitive weakness or impairment

Flexible algorithms for identifying a developmental cognitive weakness or acquired cognitive impairment on the fluid tests were developed (Table 3). Some algorithms consist of a specific pattern of low scores, whereas other algorithms refer to whether a child or adolescent displays one of multiple possible patterns of low scores. For example, a potential algorithm to identify a developmental weakness or acquired cognitive impairment for youth with average crystalized ability (SS = 100–109/T = 50–56) is having two or more scores ≤ 16th percentile. The base rate for this pattern of low scores is 14.2% for age-adjusted norms and 12.6% for demographically adjusted norms. Another potential algorithm to identify a cognitive weakness or impairment for youth with the same crystalized ability is whether an examinee obtains three or more scores ≤ 25th percentile *or* two or more scores ≤ 9th percentile. The base rate for having either of these patterns of low scores is 11.5% for age-adjusted norms and 10.6% for demographically adjusted norms.

**Table 3:** Flexible algorithms for identifying cognitive weakness or impairment on the NIHTB-CB fluid subtests for children and adolescents ages 7–17.

			Age-adjusted norms		Demographically-adjusted norms	
Comparison group	12 algorithms		Cutoff base rate	Overall base rate	Cutoff base rate	Overall base rate
Full sample (ages 7–17)	1)	4+ scores ≤ 25th, or	4.4%	9.7%	4.3%	7.9%
		3+ scores ≤ 16th, or	5.0%	–	5.4%	–
		2+ scores ≤ 9th	6.8%	–	4.4%	–
	2)	3+ scores ≤ 25th	13.3%	13.3%	14.9%	14.9%
	3)	2+ scores ≤ 16th	16.8%	16.8%	17.1%	17.1%
Crystalized composite SS ≤ 89/T ≤ 43 (low average)	4)	5 scores ≤ 25th, or	2.0%	7.3%	1.3%	6.2%
		4+ scores ≤ 16th, or	3.0%	–	2.3%	–
		3+ scores ≤ 9th, or	3.7%	–	2.0%	–
		2+ scores ≤ 5th	5.0%	–	4.2%	–
	5)	3+ scores ≤ 16th	10.0%	10.0%	8.5%	8.5%
	6)	2+ scores ≤ 9th	13.3%	13.3%	9.1%	9.1%
Crystalized composite SS = 90–99/T = 44–49 (average)	7)	4+ scores ≤ 25th, or	5.6%	11.1%	5.7%	9.3%
		3+ scores ≤ 16th, or	4.3%	–	7.3%	–
		2+ scores ≤ 9th	6.9%	–	4.3%	–
	8)	3+ scores ≤ 25th	15.7%	15.7%	18.0%	18.0%
	9)	2+ scores ≤ 16th	17.7%	17.7%	19.0%	19.0%
Crystalized composite SS = 100–109/T = 50–56 (average)	10)	3+ scores ≤ 25th, or	10.6%	11.5%	10.3%	10.6%
		2+ scores ≤ 9th	5.0%	–	2.7%	–
	11)	2+ scores ≤ 16th	14.2%	14.2%	12.6%	12.6%
Crystalized composite SS ≥ 110/T ≥ 57 (above average)	12)	3+ scores ≤ 25th, or	7.1%	11.1%	9.2%	14.2%
		2+ score ≤ 16th	9.3%	–	11.7%	–

NIHTB-CB, National Institutes of Health Toolbox for the Assessment of Neurological and Behavioral Function Cognition Battery. Age norms are provided as age-corrected standard scores (SS; M = 100, SD = 15) and fully demographic corrected norms are provided as T scores (M = 50, SD = 10) adjusted for age, sex, maternal education level, and race/ethnicity. The NIHTB-CB includes five tests of fluid abilities: Flanker Inhibitory Control and Attention Test, Picture Sequence Memory Test, List Sorting Working Memory Test, Dimensional Change Card Sort Test, and Pattern Comparison Processing Speed Test. Two tests measure crystalized abilities, from which the crystalized composite is calculated: Picture Vocabulary Test and Oral Reading Recognition Test.

## 4 Discussion

It was common for children and adolescents in the standardization sample to obtain at least one low score across the five NIHTB-CB fluid subtests. Across the NIHTB-CB fluid subtests, nearly two-thirds of children and adolescents obtained one or more scores ≤ 25th percentile, half obtained one or more scores ≤ 16th percentile, between a third and a fourth obtained one or more scores ≤ 9th percentile, and nearly a fifth obtained one or more low scores ≤ 5th percentile. This finding is consistent with numerous prior studies on pediatric normative and standardization samples for various test batteries (Brooks, 2010; Brooks and Iverson, 2012; Brooks et al., 2009, 2010a, 2010b, 2013b; Cook et al., 2019). It is well established that low scores are more frequent when more tests are administered and interpreted. In the full sample, the base rate of obtaining one or more low scores ≤ 5th percentile on the five fluid NIHTB-CB tests is about 16% (age-adjusted norms: 15.9%; demographically adjusted norms: 16.3%). In comparison, the frequency of obtaining one or more low scores ≤ 5th percentile considering six subtests from the Children’s Memory Scale is 22.4% (Brooks et al., 2009) and considering the four index scores from the Wechsler Intelligence Scale, Fourth edition (WISC-IV) is 14.6% (Brooks, 2010). Also consistent with prior studies, the number of low scores obtained is related to the cutoff used to define a low score. For example, when low scores were defined as falling ≤ 25th percentile, 62.4% of youth obtained at least one low age-adjusted NIHTB-CB fluid score, but only 7.1% obtained one or more low scores ≤ 2nd percentile.

It is important to note that we computed the base rates of low scores and the algorithms using only youth who had no reported neurodevelopmental, psychiatric, or medical conditions, and thus they represent the healthiest sample of youth included in the overall NIHTB normative sample. Given that we analyzed data from the normative sample, the percentage of youth obtaining a low score on each test would be relatively close to the percentile rank (i.e., approximately 16% would obtain a score at or below the 16th percentile). However, given that we excluded youth from the normative sample who had preexisting conditions, this would mean that the percentage of included participants scoring below the percentile cutoff for each test was somewhat lower than the percentile cutoff would suggest due to excluding children and adolescents who, on average, potentially scored lower on the NIHTB-CB. Nonetheless, our results highlight the important finding that the frequency of obtaining one or more low scores across a battery (so called multivariate base rates) differ markedly from the expected frequency for any given test considered in isolation.

Estimated overall intellectual ability (i.e., the crystalized composite scores) was strongly associated with the frequency of low NIHTB-CB fluid test scores. For example, nearly two-thirds of youth (i.e., 62.8%) with a low average crystalized composite (i.e., SS ≤ 89) obtained at least one score at or below the 16th percentile, compared to one-third of youth (i.e., 33.6%) with a high average crystalized composite (i.e., SS ≥ 110). Numerous prior studies have found that intellectual ability is associated with the likelihood of obtaining low scores across pediatric test batteries (Brooks, 2010, 2011; Brooks et al., 2009; Cook et al., 2019). Of note, in a study of low scores among an inpatient pediatric psychiatric sample, estimated intellectual ability was the only independent predictor of low scores (Gaudet et al., 2019).

### 4.1 Algorithms for identifying developmental cognitive weakness or acquired cognitive impairment

The flexible algorithms presented in Table 3 can be used to identify potential cognitive weakness or impairment. For example, when using age-corrected norms based on the full sample, there are two options that classify around 16% of the sample as having possible cognitive weakness or impairment. For the first option (Algorithm #1), there are multiple successive cutoffs. If a child obtains four or more scores ≤ 25th percentile, that would qualify as cognitive weakness or impairment. If not, a clinician or researcher would apply the next cutoff: three or more scores ≤ 16th percentile. If that criterion is met, that would qualify as cognitive weakness or impairment. If not, the third and final criterion would be applied: two or more scores ≤ 9th percentile. Among the children and adolescents from the NIHTB-CB standardization sample included in this study, only 9.7% met at least one of these criteria and thus could be classified showing cognitive weakness or impairment on the fluid tests using Algorithm #1 (see Table 3). For the second option (Algorithm #2), there is only one criterion: three or more scores ≤ 25th percentile, with a base rate of 13.3% of children and adolescents in this sample.

There are also algorithms provided for youth stratified by their NIHTB-CB crystalized composite. For example, there are two options (Algorithm #10 and Algorithm #11) provided for those with crystalized abilities in the average range (i.e., age-adjusted SS = 100–109 or demographically adjusted T = 50–56). Considering the algorithms for demographically adjusted scores, cognitive weakness or impairment could be identified if (a) the examinee obtains three or more scores ≤ 25th percentile or, if not, the examinee obtains two or more scores ≤ 9th percentile (Algorithm #10); or (b) the examinee obtains two or more scores ≤ 16th percentile (Algorithm #11). For Algorithm #10, 10.6% of children and adolescents from the NIHTB-CB normative sample had one of the two performance patterns. For Algorithm #11, 12.6% of the NIHTB-CB normative sample had this performance pattern.

It is important to note that these algorithms are meant to be selected *a priori* and then applied to interpret the results. This is critical to avoid inflating the false positive rate. For example, assume a child obtained a crystalized composite score of 99. As shown in Table 2, it is *Uncommon* for children with age-adjusted crystalized composites between 90 and 99 to obtain (a) 4+ scores ≤ 25th percentile (BR = 5.6%), (b) 3+ scores ≤ 16th percentile (BR = 4.3%), and (c) 2+ scores ≤ 9th percentile (BR = 6.9%). However, per Algorithm #7 in Table 3, the likelihood of an examinee obtaining *any* of these three performance patterns is 11.1%. If a clinician or researcher were to apply each of these performance patterns in succession, rather than selecting and applying one criterion *a priori,* the base rate and, therefore the probability of false-positive findings, would be considerably higher than any of the individual performance patterns applied in isolation.

### 4.2 Clinical implications

These results align with many prior studies demonstrating that the likelihood of a child or adolescent obtaining a low score is not equivalent to the percentile rank for that score. That is, a score at the 9th percentile on one of the NIHTB-CB fluid subtests indicates that only 9% of individuals score at that level or lower when administered just that test; but the likelihood of a child or adolescent obtaining at least one score at or below the 9th percentile when administered all five fluid subtests is 29.1% (i.e., nearly one third of the normative sample using age-adjusted norms). Base rates help reduce overinterpretation of isolated low scores, and false positive classifications of cognitive weakness, by considering that low scores are common among youth in the general population, in the absence of any illness, injury, or disorder.

The clinical importance of multivariate base rates is highlighted when considering clinical diagnoses that consider cognitive test results in their criteria, such as the DSM-5 Mild Neurocognitive Disorder, which designates neuropsychological test performances 1–2 *SDs* below the normative mean (i.e., between the 3rd and 16th percentiles) as indicative of a modest impairment in cognitive functioning (American Psychiatric Association, 2013). Applying those criteria to the NIHTB-CB five fluid subtests, 45.1% (using age-adjusted norms) to 47.1% (using demographically adjusted norms) of children and adolescents from the normative sample would meet the psychometric criteria for mild neurocognitive disorder, which is clearly an unacceptably high false positive rate.

Conversely, base rates help reduce false negatives by considering unusual performance patterns that otherwise might be considered normal. For example, obtaining three or more scores within a conventional *low average* classification range (i.e., 10th–25th percentile) is *Uncommon* for children and adolescents of high average crystalized ability, occurring in fewer than 10% of the normative sample at this ability level. Some clinicians may consider these individual scores as broadly normal, but in aggregate, they might reflect an unusual pattern of performance potentially indicative of a decline from prior levels of cognitive functioning (in the appropriate clinical context, such as after a moderate traumatic brain injury or a critical illness requiring hospitalization or intensive care).

It is important to carefully consider factors that might be associated with obtaining low scores, as opposed to relying fully on multivariate base rates to guide an interpretation of low individual scores or low patterns of performances. Examinees might obtain low scores on the NIHTB-CB due to such factors as a longstanding developmental cognitive weakness, an acquired deficit due to a brain injury or medical condition, inconsistent or suboptimal effort, misunderstanding test instructions, sacrificing speed for accuracy (e.g., on the Pattern Comparison test), fatigue during testing, or anxiety associated with testing.

In contrast, it is possible that a single low test score might represent an area of clinical focus, particularly if there is evidence of associated functional difficulties in daily life. Thus, though a single isolated low score might not provide strong evidence of cognitive impairment *per se*, it might still reflect an area of weakness amenable to some form of accommodation, intervention, or remediation efforts. Thus, clinicians are encouraged to consider base rate information in the context of other sources of data to include information about whether and how clinical conclusions drawn from base rate information correlate with everyday functioning.

The base rates and algorithms reported in this study are designed to supplement clinical interpretation and require an appreciation of additional sources of clinical information to inform a diagnosis of cognitive impairment. Low test scores alone do not define cognitive impairment, and should be interpreted in addition to observations, developmental history, medical history, neuroimaging (if available), and presenting clinical concerns. Neither the base rates, nor the algorithms, definitively establish the presence or absence of a clinical condition, loss of functioning due to an injury or illness, or clinically significant problems that require intervention. Like other normative and psychometric information, these base rates are provided to strengthen the scientific basis of test score interpretation in the context of other information and details regarding a specific case (Brooks and Iverson, 2012).

### 4.3 Limitations

The reported base rates apply to the interpretation of all five fluid tests among participants administered all seven NIHTB-CB subtests (i.e., five fluid and two crystalized tests). If fewer subtests are administered in practice, these base rates and algorithms will not be applicable. These base rates also represent point estimates of cumulative percentages based on the normative sample. Clinicians and researchers applying these percentages to the interpretation of cognitive test performances should be mindful of uncertainty surrounding these estimates, as is typical for any individual cognitive test score interpretation. This uncertainty would be especially true for stratifications with smaller sample sizes, as opposed to estimates for the full normative sample.

There are longstanding conceptual and psychometric limitations and concerns relating to cognitive profile analyses, such as scatter, strengths and weaknesses, and discrepancy scores in school psychology (McGill et al., 2018) that also are relevant and important for clinical neuropsychology. Principles from evidence-based medicine can be used to mitigate some of these conceptual and psychometric limitations, such as tying assessment data directly to clinical conclusions or decision-making using Bayesian methods (Youngstrom, 2013)—an approach that can be applied when considering multivariate base rates and clinical algorithms, such as those provided in the present study.

Another limitation pertains to the crystalized composite. The crystalized composite may serve as an estimate of “premorbid” (i.e., longstanding) functioning when premorbid testing data is unavailable. Performance on the crystalized composite is relatively resistant to the effects of brain injury (Tulsky et al., 2017), but it is not impervious to the effects of neurological injury, considering verbal intelligence can be reduced in acute and chronic pediatric brain injury (Babikian and Asarnow, 2009). Therefore, when evaluating children with acquired injuries to the brain it is important to appreciate that their obtained crystalized composite scores might not closely correspond with their premorbid functioning. This is especially important to consider for children whose obtained crystalized composite falls on the border between classification ranges. For example, imagine an adolescent who sustained a moderate traumatic brain injury 1 month prior to completing the NIHTB-CB. If that youth obtained a crystalized composite score of 109, the clinician might choose to assume that the adolescent’s premorbid (i.e., pre-injury) score was more likely to be 110 or greater, and thus apply base rate comparisons, or algorithms, based on having estimated longstanding intellectual abilities in the above average, not the average, classification range. Of note, however, stratifying base rates using a measure of crystalized intelligence (as defined by the NIHTB-CB with measures of receptive vocabulary and single word reading) may underestimate the abilities of English language learners (Ortiz, 2019) and youth with certain neurodevelopmental disorders, such as reading disorder (i.e., dyslexia) and language disorder (de Bree et al., 2022; Gray et al., 2019).

## 5 Conclusion and future directions

This study provides multivariate base rates of low scores for children and adolescents on the NIHTB-CB. It also provides flexible diagnostic algorithms that may be useful for clinicians and researchers seeking criteria to operationalize cognitive impairment. These algorithms can be helpful when evaluating patients and participants of varying levels of intellectual ability, allowing for different operational definitions of cognitive impairment based on crystalized ability. They may also have value when examining individuals of diverse cultural backgrounds, with algorithms based on demographically adjusted norms that consider age, gender, race/ethnicity, and maternal education as a proxy of socioeconomic status. The goal of such demographically adjusted algorithms would be to improve the accuracy of neuropsychological assessment and test score interpretation of minoritized groups. The algorithms reported in this study would benefit from validation within clinical samples to determine whether an individual classified as having cognitive impairment is more likely to have a clinical condition, functional impairment, or evidence of structural or functional changes on neuroimaging.

## Data Availability

The datasets analyzed for this study are publicly available (Gershon, 2016).
